# Robust adaptive filtering using recursive weighted least squares with combined scale and variable forgetting factors

**DOI:** 10.1186/s13634-016-0341-3

**Published:** 2016-03-31

**Authors:** Branko Kovačević, Zoran Banjac, Ivana Kostić Kovačević

**Affiliations:** 1 School of Electrical Engineering, University of Belgrade, Bulevar kralja Aleksandra 73, Belgrade, Serbia; 2 School of Electrical and Computer Engineering, 283 Vojvode Stepe St., Belgrade, Serbia; 3 Faculty of Informatics and Computing, Singidunum University, 32 Danijelova St., Belgrade, Serbia

**Keywords:** Adaptive filtering, FIR filtering, M robust parameter estimation, Recursive weighted least squares, Variable forgetting factor, Recursive variance estimation, Non-stationary noise, Impulsive noise

## Abstract

In this paper, a new adaptive robustified filter algorithm of recursive weighted least squares with combined scale and variable forgetting factors for time-varying parameters estimation in non-stationary and impulsive noise environments has been proposed. To reduce the effect of impulsive noise, whether this situation is stationary or not, the proposed adaptive robustified approach extends the concept of approximate maximum likelihood robust estimation, the so-called M robust estimation, to the estimation of both filter parameters and noise variance simultaneously. The application of variable forgetting factor, calculated adaptively with respect to the robustified prediction error criterion, provides the estimation of time-varying filter parameters under a stochastic environment with possible impulsive noise. The feasibility of the proposed approach is analysed in a system identification scenario using finite impulse response (FIR) filter applications.

## Introduction

Adaptive filtering represents a common tool in signal processing and control applications [1–6]. An overview of methods for recursive parameter estimation in adaptive filtering is given in the literature [5–7]. There is, unfortunately, no recursive parameter estimation that is uniformly best. Recursive least squares (RLS) algorithm has been applied commonly in adaptive filtering and system identification, since it has good convergence and provides for small estimation error in stationary situations and under assumption that the underlying noise is normal [5–7]. In this context, however, two problems arise.

First, in the case of time varying parameters, forgetting factor (FF) can be used to generate only a finite memory, in order to track parameter changes [7, 8]. For a value of FF smaller than one, one can estimate the trend of non-stationarity very fast but with higher estimate variance, owing to smaller memory length. On the other hand, with a FF close to unity, the algorithm has wider memory length and needs rather a relatively long time to estimate the unknown coefficients. However, these coefficients are estimated accurately in stationary situations. Moreover, RLS with fixed value of FF (FFF) is not effective for tracking time-varying parameters with large variations. This makes it necessary to incorporate an adaptive mechanism in the estimator, resulting in the concept of variable FF (VFF). Several adaptation procedures have been discussed by changing the memory length of signal [7–13]. In particular, the methods referred as the parallel adaptation RLS algorithm (PA-RLS) and extended prediction error RLS-based algorithm (EPE-RLS) have a good adaptability in non-stationary situations [9–13]. In addition, both methods assume that the variance of interfering noise is known in advance.

The second problem arises in an application where the required filter output is contaminated by heavy tailed distributed disturbance, generating outliers [14–20]. Namely, the classical estimation algorithms optimise the sum of squared prediction errors (residuals) and, as a consequence, give the same weights to error signals, yielding a RLS type procedure. However, an adequate information about the statistics of additive noise is not included in RLS computation. Possible approaches to robust system identification introduce a non-linear mapping of prediction errors. Although in the statistical literature, there are few approaches to robust parameter estimation, M robust approach (the symbol M means approximate maximum-likelihood) is emphasised, due to its simplicity for practical workers [15–23]. Robustified RLS algorithm, based on M robust principle, the so-called robustified recursive least square method (RRLS), uses the sum of weighted prediction errors as the performance index, where the weights are functions of prediction residuals [24–26]. However, M estimators provide for the solutions of the location parameters estimation problem [21–23]. As a consequence, in a situation of non-stationary noise signal with the time-varying variance, their efficiency should be bad [7, 24]. Therefore, a significant part of RRLS algorithm is the estimation of unknown noise variance or the so-called scale factor [15, 16, 24, 26]. A suitable practical robust solution is the median estimator based on absolute median deviations, named median of absolute median deviations (MAD) estimator [21–23]. However, RRLS algorithm of M robust type using MAD scale factor estimation is also found to be non-effective for tracking of time-varying parameters [7, 11, 24, 26]. For these reasons, neither of the stated algorithms alone can solve the both mentioned problems.

In this article, we design a new robust adaptive finite impulse response (FIR) system for dealing with these problems simultaneously. To alleviate the effects of non-stationary and impulsive noise, this algorithm extends the concept of M robust estimation to adaptive M robust algorithm with the estimation of both filter parameters and unknown noise variance simultaneously. The estimated noise variance, together with the robustified extended prediction error criterion, calculated on the sliding data frame of proper length, is used to define a suitable robust discrimination function, as a normalised measure of signal non-stationarity. In addition, the VFF is introduced by the linear mapping of robust discrimination function. This, in turn, enables the tacking of time-varying filter parameter under the impulsive noise. Simulation results demonstrate the effectiveness of the proposed algorithm, by the comparison with the conventional recursive least squares (RLS) using VFF based on the standard EPE criterion, and the adaptive M robust-based algorithm with only scale factor (RRLS).

## Problem formulation

**Fig. 1:**
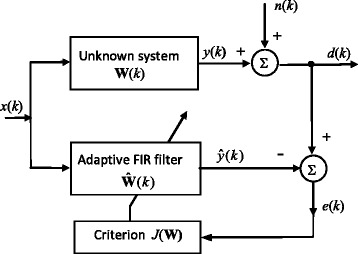
System identification configuration

A commonly used adaptive FIR filtering scenario, expressed as a system identification problem, is presented in Fig. 1. Here, *x*(*k*) is the random input signal, *d*(*k*) is the required filter output, *n*(*k*) is the noise or disturbance and *e*(*k*) is the prediction error or residual. The filter parameter vector, **W**(*k*), can be estimated recursively by optimising the prespecified criterion. The RLS algorithm with FF approaches the problem of estimation of non-stationary (time-varying) signal model parameters by minimising the sum of exponentially weighted squared residuals [5–7, 25, 26]. On the other hand, robust estimates are insensitive to outliers, but are inherently non-linear. Moreover, most robust regression procedures are minimization problems [15–26]. Specifically, M robust estimates are derived as minimization of the sum of weighted residuals, instead of the quadratic performance criterion in the classical RLS computation [21–23]. To combine these two approaches, let us define a new criterion as the sum of weighted residuals

1 $$ {J}_k\left(\mathbf{W},s\right)=\frac{1}{k}{\displaystyle \sum_{i=1}^k{\rho}^{k-i}\varphi \left(\frac{e\left(i,\mathbf{W}\right)}{s}\right)} $$

where the prediction error (residual) signal is given by

2 $$ e\left(i,\mathbf{W}\right)=d(i)-{\mathbf{X}}^T(i)\mathbf{W} $$

with the regression vector, **X**(*i*), and the parameter vector, **W**, being defined by

3 $$ \mathbf{X}(k)={\left[x(k),\kern0.36em x\left(k-1\right), \dots \kern0.24em ,\;x\left(k-n+1\right)\right]}^T;\kern0.36em \mathbf{W}={\left[{w}_1,{w}_2,\cdots, {w}_n\right]}^T $$

Here, the quantities *s* and *ρ* represent the scale and forgetting factors, respectively. Moreover, **W** is the unknown filter parameter vector that has to be estimated. In addition, *φ*(⋅) is a robust score, or loss, function, which has to suppress the influence of impulsive noise, generating outliers. Having in mind the importance of reducing the influence of outliers contaminating the Gaussian noise samples, *φ*(⋅) should be similar to a quadratic function in the middle, but it has to increase more slowly in the tails than the quadratic one. In addition, its first derivative, *ψ*(⋅) = *φ*′(⋅), the so-called influence function in the statistical literatures, has to be bounded and continuous [21–23, 25, 26]. The first property provides that single outlier will not have a significant influence, while the second one provides that patchy or grouped outliers will not have a big impact. A possible choice, for example, is the Huber's robust loss function, with the corresponding influence function [21].

4 $$ \psi (x)= \min \left\{\frac{\left|x\right|}{\sigma^2},\frac{\varDelta }{\sigma}\right\}\operatorname{sgn}(x) $$

Here, sgn(⋅) is the signum function, *σ* is the noise standard deviation and *Δ* is a free parameter. This parameter can be adopted in such a way to provide for required efficiency robustness under the zero-mean white normal noise model [21–23]. The non-linear transformation of data based on (4) is known in the statistical literature as winsorization [21–23].

Taking the first partial derivate of (1) with respect to the elements of **W** in (3), say **W**_*j*_, *j* = 1, 2, …, *n*, being equal to zero, we see that the minimization of (1) reduces to finding the solution of *n* non-linear algebraic relations:

5 $$ {\displaystyle \sum_{i=1}^k{\rho}^{k-i}{\mathbf{X}}_{ij}\;\psi \left(\frac{e\left(i,\mathbf{W}\right)}{s}\right)}=0,\kern0.24em j=1,2,\dots, n $$

where **X**_*ij*_ is the element in the *j*th column of the row vector **X**^*T*^(*i*) in (3), while *ψ*(⋅) is the first derivative of *φ*(⋅), *ψ*(⋅) = *φ*^'^(⋅). Of course, for non-linear *ψ*(⋅), (5) must be solved by iterative numerical methods, and two suitable procedures are Newton-Raphson’s and Ditter’s algorithms, respectively, [27, 28]. Here, a slightly different approach is proposed using a weighted least-squares (WLS) approximation of (5). In this approach, the relation (5) is replaced by the following approximation

6 $$ {\displaystyle \sum_{i=1}^k{\mathbf{X}}_{ij}\;\beta \left(k,i\right)e\left(i,\mathbf{W}\right)}\approx 0,\kern0.24em j=1,2,\dots, n $$

where the exponentially weighted robust term is given by

7 $$ \beta \left(k,i\right)={\rho}^{k-i}\omega \left(i,{\mathbf{W}}_0\right) $$

while its robust part is defined by

8 $$ \omega \left(i,{\mathbf{W}}_0\right)=\left\{\begin{array}{c}\hfill \frac{\psi \left(\frac{e\left(i,{\mathbf{W}}_0\right)}{s}\right)}{\frac{e\left(i,{\mathbf{W}}_0\right)}{s}}\kern0.84em  if\;d(i)\ne {\mathbf{X}}^T(i){\mathbf{W}}_0\kern0.24em \mathrm{and}\kern0.24em s\ne 0\hfill \\ {}\hfill \kern1.92em 1\kern3.84em  if\kern0.24em d(i)={\mathbf{X}}^T(i){\mathbf{W}}_0\kern0.24em \mathrm{and}/\mathrm{or}\kern0.24em s=0\kern0.48em \hfill \end{array}\right. $$

Here, **W**_0_ is an initial estimate of the parameter vector, **W**, which can be obtained, for example, by using the conventional non-recursive LS estimator [5, 25, 26]. The solution of (6), say **Ŵ**(*k*), represents a one-step non-recursive suboptimal M robust estimate of **W** in (3).

Application of the non-recursive M robust scheme (6)–(8) requires the non-linear residual transformation, *ψ*(⋅), and scaling factor, *s*, to be defined in advance. But, in general, the standard deviation, *σ*, in (4) is not known beforehand and has to be estimated somehow. A commonly used robust estimate of *σ* in the statistical literature is the median scheme, based on the absolute median deviations [21–23]

9 $$ s=\frac{\mathrm{median}\left|{e}_i-\mathrm{median}\left({e}_i\right)\;\right|}{0.6745},\kern0.72em i=1,2,\dots, L $$

where *L* denotes the length of sliding data frame. The divisor 0.6745 in (8) is used because the MAD scale factor estimate, *s*, is approximately equal to the noise standard deviation, *σ*, if the sample size, *L*, is large and if samples actually arise from a normal distribution [21]. Moreover, because *s* ≈ *σ*, *Δ* is usually taken to be 1.5 [21–23]. This choice will produce much better results, in comparison to the RLS method, when the corresponding noise probability density function (pdf) has heavier tails than the Gaussian one. Furthermore, it will remain good efficiency of RLS when the pdf is exactly normal [21–23].

The weighting term, *ω*, in (8) is not strictly related to the popular robust MAD estimation of the scale factor, *s*. This estimate guarantees that *s* ≠ 0, but in the general case, a scale factor estimator may not guarantee that the estimate, *s*, should become equal to zero. This is the reason why the condition *s* = 0 is included in (8). Particularly, the application of a recursive robustified scale factor estimation requires the initial guess, *s*(0), to be given beforehand. A common choice is *s*(0) = 0, but in the first few steps, the obtained estimate of scale factor can be equal to zero, so that the unit value of *ω* in (8) has to be chosen.

The proposed suboptimal M robust estimator (6)–(8) is numerically simpler than the ones oriented towards solving the non-linear optimization problem in (5), but it still remains complex computation. Namely, this method does not have an attractive recursive form and, therefore, is not computationally feasible as the RLS type estimators. Moreover, M robust approach is conservative and may degrade without further adaptation [15–20]. Starting from the proposed non-recursive M robust estimator (6)–(8), a simple and practically applicable recursive M robust parameter estimation procedure with both adaptive robustified scale and variable forgetting factors is derived in the next paragraph. Some alternative approaches for the scale factor adaptation can be found in the literature [15, 16, 24]. Moreover, the application of the EPE-based VFF for solving different practical problems is also discussed in the literature [12, 17–20, 29, 30]. However, similarly to sample mean and sample variance, the standard EPE approach is non-robust towards outliers [21–23]. Therefore, in the next chapter, an alternative M robust approach for generating VFF adaptively is proposed.

## A new recursive robust parameter estimation algorithm with combined scale and forgetting factors

The solution of (6) can be also represented in the computationally more feasible recursive form, using the well-known algebraic manipulations (for more details, see Appendix 1), [25, 26]. This results in the parameter estimation algorithm

10 $$ \widehat{\mathbf{W}}(k)=\widehat{\mathbf{W}}\left(k-1\right)+\mathbf{K}(k)e\left(k,\widehat{\mathbf{W}}\left(k-1\right)\right) $$

11 $$ e\left(k,\widehat{\mathbf{W}}\left(k-1\right)\right)=d(k)-{\mathbf{X}}^T(k)\widehat{\mathbf{W}}\left(k-1\right) $$

12 $$ \mathbf{K}(k)=\frac{\mathbf{M}(k)\mathbf{X}(k)\omega (k)}{1+{\mathbf{X}}^T(k)\mathbf{M}(k)\mathbf{X}(k)\omega (k)};\kern0.6em \mathbf{M}(k)=\frac{1}{\rho}\mathbf{P}\left(k-1\right) $$

13 $$ \mathbf{P}(k)=\left(\mathbf{I}-\mathbf{K}(k){\mathbf{X}}^T(k)\right)\mathbf{M}(k) $$

Here, the term *ω*(*k*) is defined by (8), when the initial estimate, **W**_0_, is replaced by the preceding estimate, **Ŵ**(*k* − 1), while the prediction error (residual) in (11) is given by (2), when the unknown parameter vector, **W**, is substituted by **Ŵ**(*k* − 1).

Application of recursive M robust estimation algorithm (10)–(13) assumes the non-linear transformation, *ψ*(⋅) in (4), as well as the scale factor, *s* in (8), and the forgetting factor, *ρ* in (12), to be known. Since the scale factor, *s*, represents an estimate of the unknown noise standard deviation, *σ*, and the argument of non-linearity *ψ*(⋅) in (8) is the normalised residual, the non-linear transformation *ψ*(⋅) in (8) is defined by (4) with the unit variance, i.e. *σ* = 1. Particularly, if one choses the linear transformation in (8), *ψ*(*x*) = *x*, this results in the unit weight, *ω*(*k*) = 1 in (8), and algorithm (10)–(13) reduce to the standard RLS algorithm with FF defined by (10) and (11), where the corresponding matrices, instead of (12) and (13), are given by [5, 25, 26]

14 $$ \mathbf{K}(k)=\mathbf{P}\left(k-1\right)\mathbf{X}(k){\left[\rho +{\mathbf{X}}^T(k)\mathbf{P}\left(k-1\right)\mathbf{X}(k)\right]}^{-1} $$

15 $$ \mathbf{P}(k)=\frac{1}{\rho}\left(\mathbf{P}\left(k-1\right)-\frac{\mathbf{P}\left(k-1\right)\mathbf{X}(k){\mathbf{X}}^T(k)\mathbf{P}\left(k-1\right)}{\;\rho +{\mathbf{X}}^T(k)\mathbf{P}\left(k-1\right)\mathbf{X}(k)}\right) $$

In addition, if one defines the Huber’s non-linearity in (4) by using the non-normalised argument on the right-hand side of (4), yielding

16 $$ \psi (x)= \min \left(\left|x\right|,\varDelta \sigma \right)\mathrm{sign}(x) $$

and approximate the first derivate, *ψ*′(⋅), by the weighted term *ω*(*x*) = *ψ*(*x*)/*x* in (8), together with the application of the winsorised residual, *ψ*(*e*), instead of original one, *e*, in the parameter update equation (10), algorithm (10)–(13) can be rewritten as

17 $$ \widehat{\mathbf{W}}(k)=\widehat{\mathbf{W}}\left(k-1\right)+\mathbf{P}(k)\mathbf{X}(k)\psi \left[e(k)\right] $$

18 $$ \mathbf{P}(k)=\mathbf{P}\left(k-1\right)-\frac{\mathbf{P}\left(k-1\right)\mathbf{X}(k){\mathbf{X}}^T(k)\mathbf{P}\left(k-1\right){\psi}^{\prime}\left[e(k)\right]}{1+{\psi}^{\prime}\left[e(k)\right]{\mathbf{X}}^T(k)\mathbf{P}\left(k-1\right)\mathbf{X}\left(k-1\right)} $$

where the prediction error (residual), *e*,is given by (11).

The obtained algorithm in (3), (11), (16)–(18), represents the standard M robust RLS (RRLS), where the common approach is to estimate the unknown noise standard deviation, *σ*, by the MAD based scale factor in (9). This algorithm can be exactly derived by applying the Newton-Raphson iterative method for solving the non-linear optimization problem in (1), with the unit parameters *s* and *ρ*, respectively. Here, the non-linearity, *ψ*, in (16) represents the first derivative of the loss function, *φ*, in (1) [24–26]. It should be noted that the parameter update equation in (17) is non-linear, in contrast to the linear parameter update equation in (10). However, both procedures for generating the weighting matrix sequences, *P*(*k*), in (12), (13), and (18), respectively, are non-linear. Later, it will be shown that the scheme for generating the weighting matrix is very important for achieving the practical robustness.

The proposed parameter estimation algorithm (10)–(13) are derived from the M robust concept that is conservative, so the quality of parameter estimates may degrade without further adaptations of *s* and *ρ* variables.

### Adaptive robustified estimation of scale factor

As an adaptive robust alternative to the non-recursive robust MAD estimate in (9), the scale factor, *s*, can be estimated simultaneously with the filter parameter vector, **W**. Namely, if $\overline{p}(n)$ is the pdf of zero-mean Gaussian white noise, *n*(*k*), in Fig. 1, with the unit variance, then the pdf of noise with some variance, *σ*^2^, is given by $p(n)=\overline{p}\left(n/\sigma \right)/\sigma$. Thus, one can define an auxiliary performance index, in the form of the conditional maximum-likelihood (ML) criterion, [25, 26].

19 $$ J\left(\sigma /\mathbf{W}\right)=E\left\{F\left(\frac{e\left(k,\mathbf{W}\right)}{\sigma}\right)/\mathbf{W}\right\};\kern0.6em F(n)=- \ln \left(p(n)\right) $$

where *e*(⋅) is the prediction error signal in (11) and *E*{⋅/**W**} represents the conditional mathematical expectation when the parameter vector, **W**, is given. Furthermore, one can use the Newton’s stochastic algorithm for recursive minimization of the performance index in (19) [25, 26].

20 $$ s(k)=s\left(k-1\right)-{\left[k\frac{\partial^2J\left(s\left(k-1\right)/\widehat{\mathbf{W}}\left(k-1\right)\right)}{\partial {\sigma}^2}\right]}^{-1}\left[k\frac{\partial J\left(s\left(k-1\right)/\widehat{\mathbf{W}}\left(k-1\right)\right)}{\partial \sigma}\right] $$

where **Ŵ**(*k*) and *s*(*k*) are the corresponding estimates, at time instant *k*, of **W** and *σ*, respectively. In addition, let us introduce the empirical approximation of the criterion (19) as

21 $$ {J}_k\left(s/\widehat{\mathbf{W}}\right)=\frac{1}{k}{\displaystyle \sum_{i=1}^kF\left(\frac{e\left(i,\widehat{\mathbf{W}}\right)}{s}\right)} $$

Under certain conditions, with *k* increasing, *J*_*k*_ in (21) approaches to *J* in (19). Moreover, since $p(n)=\overline{p}\left(n/\sigma \right)/\sigma$, one obtains from (19)

22 $$ F(n)= \ln \left(\sigma \right)+f\left(\frac{n}{\sigma}\right);\kern0.6em f\left(\frac{n}{\sigma}\right)=- \ln \left(\overline{p}\left(\frac{n}{\sigma}\right)\right) $$

In addition, with large *k* and by using the optimality conditions, yielding

23 $$ \frac{\partial J\left(s/\widehat{\mathbf{W}}\right)}{\partial \sigma}\approx \frac{\partial {J}_k\left(s/\widehat{\mathbf{W}}\right)}{\partial s};\kern0.24em \frac{\partial^2J\left(s/\widehat{\mathbf{W}}\right)}{\partial {\sigma}^2}\approx \frac{\partial^2{J}_k\left(s/\widehat{\mathbf{W}}\right)}{\partial {s}^2};\kern0.36em {J}_{k-1}\left(s/\widehat{\mathbf{W}}\right)\approx 0 $$

one obtains from (20)–(23) an approximate optimal solution in the recursive form

24 $$ ks(k)=\left(k-1\right)s\left(k-1\right)+e\left(k,\widehat{\mathbf{W}}\left(k-1\right)\right)g\left(e\left(k,\widehat{\mathbf{W}}\left(k-1\right)\right)/s\left(k-1\right)\right) $$

or equivalently

25 $$ k{s}^2(k)=\left(k-1\right){s}^2\left(k-1\right)+{e}^2\left(k,\widehat{\mathbf{W}}\left(k-1\right)\right)\omega (k) $$

Here, the robust weighting term, *ω*(*k*), is defined by (8), when *ψ* function is changed by *g* function, while **W**_0_ is substituted by **Ŵ**(*k* − 1) and *s* by *s*(*k* − 1), respectively. As mentioned before, in M robust estimation, we wish to design estimators that are not only quite efficient in the situations when the underlying noise pdf is normal but also remain high efficiency in situations when this pdf possesses longer tails than the normal one, generating the outliers [21–27]. Thus, we can define M robust estimator not exactly as the ML estimator based on the standard normal pdf $\overline{p}(n)$, with zero-mean and unit variance, but ML estimator corresponding to a pdf $\overline{p}(n)$ that is similar to the standard Gaussian pdf in the middle, but has heavier tails than the normal one. This corresponds, for example, to the double exponential, or the Laplace pdf. Such choice corresponds further to the *f*(⋅) function in (22) being equal to the Huber’s M robust score function, *φ*(⋅), in (1), with the first derivative, *ψ*(⋅) = *φ* ' (⋅), given by (4), [21–23]. Thus, in this case, *g*(⋅) = *f* ' (⋅) reduces to the *ψ* -function in (4), for which the noise standard deviation, *σ*, is equal to one. In addition, *ω*(*k*) in (25) is given by (8), with **W**_0_ and *s* being equal to **Ŵ**(*k* − 1) and *s*(*k* − 1), respectively. Furthermore, when the pdf, $\overline{p}(n)$, is the standard normal, the influence function *g*(⋅) is linear and the weighting term *ω*(*k*) in (25) becomes equal to one [13]. Finally, the application of the recursive algorithm (24) or (25) requires the initial guess *s*(0) to be given beforehand, as it is done in Eq. (37).

The net effect is to decrease the consequence of large errors, named outliers. The estimator is then called robust. In algorithm (24) or (25), this goal is achieved through the weighting term in (8), where *ψ* is the saturation type non-linearity in (4). Thus, the function *ψ*(⋅) is linear for small and moderate arguments, but increases more slowly than the liner one for large arguments. Furthermore, in the normal case without outliers, one should want most of the arguments of the *ψ*(⋅) function to satisfy the inequality |*e*(*i*, **W**_0_)| ≤ *Δs*, because then *ψ*(*e*(*i*, **W**_0_)/*s*) = *e*(*i*, **W**_0_)/*s* and *ω* in (8) is equal to unity. On the other hand, for large arguments satisfying |*e*(*i*, **W**_0_)| > *Δs*, the weighting term in (8) decreases monotonously with the argument absolute value and, as a consequence, reduces the influence of outliers.

For the scale estimation problem in question, the unknown noise variance is assumed to be constant. Therefore, after time increases, the derived recursive estimates (24) or (25) converge towards the constant value. Equations (24) and (25) represent the linear combination of the previous estimate and the robustly weighted current estimation error. The coefficients in the linear combination, 1 − 1/*k* and 1/*k*, depend on the time step, *k*. Thus, as *k* increases, these coefficients converge towards unity and zero, respectively. As a consequence, after a sufficiently large time step, *k*, the correcting term in (24) and (25) that is multiplied by the coefficient 1/*k* is close to zero, so that the proposed algorithm eliminates the effect of possible outliers.

Moreover, in many practical problems, it is of interest to consider the situation in which the noise variance is time-varying. However, due to the described saturation effect, the proposed estimator cannot catch the changes. These situations can be covered by simple extension of Eqs. (24) and (25). A simple but efficient solution can be obtained by resetting. The forgetting or discounting factor, 1/*k*, in (24) and (25) is then periodically reset to the unit value, for example each 100 steps, and the initial guess, *s*(0), has to be set to the previous estimate.

### Strategy for choosing adaptive robustifying variable forgetting factor

As mentioned before, the value of forgetting factor FF, *ρ*, belongs to the set of real numbers (0,1], as it has to give more heavily weights to the current samples, in order to provide for tracking of time-varying filter parameters. If a value of FF, *ρ*, is close to one, it needs rather long time to find the true coefficients. However, the parameter estimates should be with high quality in stationary situations. The speed of adaptation can be controlled by the asymptotic memory length, defined by [7, 25].

26 $$ N=\frac{1}{1-\rho } $$

Thus, it follows from (26) that progressively smaller values of FF, *ρ*, provides an estimation procedure with smaller size of data window, what is useful in non-stationary applications.

If a signal is synthetised of sub-signals having different lengths of memory, changing between a minimum value, *N*_min_, and a maximum one, *N*_max_, the time-varying signal model coefficients can be estimated by using Eqs. (4), (8), (10–13), and (25), assigning to each sub-signal the corresponding FF, *ρ*, from (26), varying between *ρ*_min_ and *ρ*_max_. However, in practice, the memory length and the starting points of sub-signals are unknown in advance. Thus, one has to find the degree of signal non-stationarity, in order to generate the value of FF, *ρ*, in the next step. Although many adaptation procedures have been analysed by changing the memory length, the method using the extended prediction error (EPE) criterion is emphasised, since it involves rather easy computation, and has good adaptability in non-stationary situations, and a low variance in the stationary one [10, 12, 13]. Particularly, the extended prediction error criterion, as a local measure of signal non-stationarity, is defined by [10]:

27 $$ E(k)=\frac{1}{L}{\displaystyle \sum_{i=k-L+1}^k{e}^2\left(i,\widehat{\mathbf{W}}\left(i-1\right)\right)} $$

Here, *e*(⋅) is the prediction error, or residual, in (11), and the length of the sliding window *L* is a free parameter, which has to be set.

Thus, the quantity *E*(*k*) in (27) represents a measure of the local variance of prediction residuals at the given sliding data frame of size *L*, and it contains the information about the degree of data non-stationarity. In addition, *L* should be a small number compared to the minimum asymptotic memory length, so that averaging does not obscure the non-stationarity of signal. Thus, the value of *L* represents the trade-off between the estimation accuracy and tracking ability of time varying parameters. Unfortunately, the EPE statistics in (27), like the sample mean and sample variance, lacks robustness towards outliers [21–23]. Therefore, we suggest to derive a robust alternative to the EPE criterion in (27) using the M robust approach. Thus, if the prediction errors *e*(*k*) in (2) are assumed to be independent and identically distributed (i.i.d) random variables, a simple parameter estimation problem can be constructed. Define a random variable (r.v.), *ζ*, on the sample space *Ω*, from which the data *e*(*k*), *k* = 1, 2, ⋯, *N*, are obtained. Based on empirical measurements, the mean, *m*_*e*_, and variance, ${\sigma}_e^2$, of the unknown distribution of r.v., *ζ*, are to be estimated. As in (1), the robust M estimate ${\widehat{m}}_e(N)$ of *m*_*e*_ is defined by

28 $$ {\displaystyle \sum_{k=1}^N\psi \left(\frac{e(k)-{\widehat{m}}_e(N)}{s}\right)}=0 $$

where *ψ*(⋅) is the Huber’s influence function in (4). Here, *s* is an estimate of the scale of the data {*e*(*k*)}. The estimating Eq. (28) is non-linear, and some form of WLS approximation, similar to (6), can be used for its solution. Moreover, a popular statistic *s* is the MAD estimation in (9). Although *s* in (9) is robust, it turns out to be less efficient than some other robust estimates of variance [21–23]. However, *s* is a nuisance parameter in the computation of *m*_*e*_, and in this context, the efficient issue is not as crucial as in the estimation of the variance of the data, estimates of the latter being used in robustifying the EPE criterion (27) and setting the VFF. A more efficient estimator of the data variance should be based on the asymptotic variance formula for the location M estimate in (28). When *s* = *σ*_*e*_, this formula is given by [21]

29 $$ \mathrm{V}=\underset{N\to \infty }{ \lim }E\left\{N{\left[{\widehat{m}}_e(N)-{m}_e\right]}^2\right\}=\frac{\sigma_e^2E\left\{{\psi}^2\left(\frac{e(k)-{m}_e}{\sigma_e}\right)\right\}}{E^2\left\{{\psi}^{\prime}\left(\frac{e(k)-{m}_e}{\sigma_e}\right)\right\}} $$

A natural estimate of V in (29) is

30 $$ {\widehat{\mathrm{V}}}_N={s}^2\frac{\frac{1}{N}{\displaystyle \sum_{k=1}^N{\psi}^2\left(\frac{e(k)-{\widehat{m}}_e(N)}{s}\right)}}{\frac{1}{N}{\displaystyle \sum_{k=1}^N{\psi}^{\prime}\left(\frac{e(k)-{\widehat{m}}_e(N)}{s}\right)}} $$

where ${\widehat{\mathrm{V}}}_N$ in (30) would appear to be a reasonable estimate of ${\sigma}_e^2$, with ${\widehat{m}}_e(N)$ being the M estimate of *m*_*e*_ in (28). Therefore, given *m*_*e*_ = 0, producing ${\widehat{m}}_e(N)=0$ and the estimate *s* from the recursive M robust scale estimate, *s*(*k*), in (25), a possible M robust alternative of the EPE criterion in (27) is given by

31 $$ {\mathrm{E}}_r(k)={s}^2(k)\frac{{\displaystyle \sum_{i=k-L+1}^k{\psi}^2\left(\frac{e\left(i,\widehat{\mathbf{W}}\left(i-1\right)\right)}{s(i)}\right)}}{{\displaystyle \sum_{i=k-L+1}^k{\psi}^{\prime}\left(\frac{e\left(i,\widehat{\mathbf{W}}\left(i-1\right)\right)}{s(i)}\right)}} $$

where *ψ*(⋅) is the Huber’s influence function in (4), with *σ* = 1 and *Δ* = 1.5. If *ψ*(⋅) is a linear function, *ψ*(*x*) = *x*, than the criterion (31) reduces to the standard EPE criterion in (27), under the assumption that the scale factor estimate, *s*(*i*), *i* = *k* − *L* + 1, ⋯, *k*, on the sliding data frame of length *L* is close to the *s*(*k*) value.

On the other hand, the total noise variance robust estimate in (25) is rather insensitive to the local non-stationary effects. Therefore, in order to make the estimation procedure invariant to the noise level, one can define the normalised robust measure of non-stationarity or the so-called robust discrimination function

32 $$ Q(k)=\frac{E_r(k)}{s^2(k)} $$

A strategy for choosing the VFF at current time instant, *k*, may now be defined by using the relations (25), (26), (31) and (32), that is

33 $$ \rho (k)=1-\frac{1}{N(k)},\kern0.6em N(k)=\frac{N_{\max }}{Q(k)} $$

Thus, the maximum asymptotic length, *N*_max_, will determine the adaptation speed. Furthermore, for a stationary signal with possible outliers, the quantity *E*_*r*_(*k*) in (31) will converge to the noise variance, yielding *Q*(*k*) ≈ 1 and *N*(*k*) = *N*_max_. Finally, since (33) does not guarantee that FF, *ρ*, does not become negative, a reasonable limit has to be placed on FF, *ρ*, yielding

34 $$ \rho (k)= \max \left\{1-\frac{1}{N(k)},\;{\rho}_{\min}\right\} $$

where *N*(*k*) is given by the relations (25) and (31)–(33).

A brief description of the proposed algorithm with both scale and forgetting factors is given in Table 1. Since the proposed algorithm combines the three adaptive procedures (recursive robust parameter estimation, recursive robustified noise variance estimation and adaptive robustified variable forgetting factor calculation), the theoretical performance analysis is very difficult with the coupled algorithms. Therefore, the figure of merit of the proposed approach will be given by simulations in the next section. The following algorithms are tested:

**Table 1:** Summary of adaptive robust parameter estimation algorithm (10)–(13) with scale factor (25) and EPE-based VFF (27)–(30)

Step 1	Let at stage *k*, *k* ≥ *N*, the parameter vector estimate **Ŵ**(*k* − 1), the scale estimates *s*(*k* − 1), …, *s*(*k* − *L* + 1), the error signals *e*(*k* − 1), …, *e*(*k* − *L* + 1) and the matrix **P**(*k* − 1) from the (*L* − 1) previous stages are known.
Step 2	Take the current input, *x*(*k*), and form the regression vector in (3) **X** ^*T*^(*k*) = {*x*(*k*), *x*(*k* − 1), …, *x*(*k* − *N* + 1)} of length *N*, assuming that the (*N* − 1) most recent inputs are given.
Step 3	Take the current output, *d*(*k*), and calculate the current error signal, *e*(*k*), from (11) using **X**(*k*) from step 2, and define the current data frame *E*_*L*_ = {*e*(*k*), *e*(*k* − 1), …, *e*(*k* − *L* + 1)} of length *L* < *N*, assuming that the (*L* − 1) most recent errors are previously stored.
Step 4	Calculate the normalised error *e*(*k*)/*s*(*k* − 1) and the winsorised error *ψ*(*e*(*k*)/*s*(*k* − 1)) from (4) with *σ* = 1; then calculate the weight *ω*(*k*) in (25) by using (8) with **W**_0_ = **Ŵ**(*k* − 1) and *s* = *s*(*k* − 1); finally, calculate the scale factor *s*(*k*) from (25).
Step 5	Define the current data frame of normalised residuals *E*_*NL*_ = {*e*(*k*)/*s*(*k*), *e*(*k* − 1)/*s*(*k* − 1), …, *e*(*k* − *L* + 1)/*s*(*k* − *L* + 1)} from steps 1, 3 and 4; then calculate the robust discrimination function, *Q*(*k*), in (28), using the data set *E*_*NL*_; finally, calculate the VFF, *ρ*(*k*), from (29) and (30).
Step 6	Calculate the winsorised error, *ψ*(*e*(*k*)/*s*(*k*)), from (4), with *σ* = 1 and by using *e*(*k*) from step 3 and *s*(*k*) from step 4; then calculate the weight, *ω*(*k*), in (8) by using (4) with **W**_0_ = **Ŵ**(*k* − 1) and *s* = *s*(*k*).
Step 7	Calculate the matrix, **M**(*k*), in (12) with *ρ* = *ρ*(*k*) from step 5; then calculate the matrix, **K**(*k*), in (12) by using **X**(*k*) from step 2 and *ω*(*k*) from step 6.
Step 8	Calculate the parameter vector update, **Ŵ**(*k*), in (10), by using *d*(*k*) and *e*(*k*) from step 3, as well as **K**(*k*) from step 7.
Step 9	Calculate the weighting matrix, **P**(*k*), in (13) by using **M**(*k*) and **K**(*k*) from step 7, together with **X**(*k*) from step 2.
Step 10	Tune the time counter, that is increase the time index, *k* ← *k* + 1, and go back to step 2.

1. Recursive robust weighted least squares type method, defined by (4), (8) and (10)–(13) together with the both recursive robust scale estimation in (25) and adaptive robustified VFF calculation in (31)–(34), denoted as RRWLSV (see Table 1).
2. Recursive least squares algorithm with exponentially weighted residuals defined by (11), (14) and (15) and VFF given by (27) and (32)–(34), denoted as RLSVF.
3. Recursive robustified least squares algorithm defined by (16)–(18) and MAD-based scale factor estimation in (9), denoted as RRLSS.

## Experimental analysis

To analyse the performances of previously discussed methods, a linear parameter estimation scenario in stationary and non-stationary contexts, related to the additive noise, is applied (see Fig. 1) [10]. The required filter output, *d*(*k*), is generated by passing the standard white Gaussian sequence, *x*(*k*), of the zero-mean and unit variance, through the FIR system of the ninth order, with the true values of parameters

35 $$ \mathbf{W}={\left[0.1,0.2,0.3,0.4,0.5,0.4,0.3,0.2,0.1\right]}^T $$

In addition, a zero-mean white additive noise, *n*(*k*), with corresponding variance is involved to its output. The value of variance is adopted so to give the desired signal-to-noise ratio (SNR) at the signal segment in question, before the impulsive noise component is introduced. Four situations regarding the additive noise are considered: stationary context with fixed variance and possible outliers and non-stationary context with changing variance and possible outliers. The variances are chosen so to give the different values of SNR equal to 15, 20 and 25 dB, respectively. The outliers, generated by the impulsive noise, are produced using the model *n*(*k*) = *α*(*k*)*A*(*k*), with *α*(*k*) being an i.i.d binary sequence defined by the corresponding probabilities *P*(*α*(*k*) = 0) = 0.99 and *P*(*α*(*k*) = 1) = 0.01, respectively, and *A*(*k*) is the zero-mean normal random variable with the variance var{*A*(*k*)} = 10^4^/12 that is independent of the random variable *α*(*k*). The random variable *n*(*k*) has zero-mean and variance proportional to var{*A*(*k*)} (see Appendix 3).

A priory information of the impulsive noise is used to choose the length, *L* = 5, of the sliding window that capture the non-stationarity. This means that no more than one outlier, in average, is in the sliding data frame of size *L* = 5 during the robustified EPE calculation in (31) and MAD calculation in (9), respectively, when the fraction of outliers is 1 %.

The following algorithms, described in previous section, are tested: (1) the proposed adaptive robust filtering algorithm with the both robust adaptive scale and VFF, denoted as RRWLSV; (2) the conventional RLS with EPE based VFF, denoted as RLSVF; and (3) the standard M robust RLS with MAD-based scale factor, denoted as RRLS.

The analysed algorithms have been tested on both the time-varying parameter tracking ability and the log normalised estimation error norm

36 $$ \mathrm{N}\mathrm{E}\mathrm{E}(k)=10 \log \frac{{\left\Vert \overset{\frown }{\mathbf{W}}(k)-\mathbf{W}\right\Vert}^2}{{\left\Vert \mathbf{W}\right\Vert}^2} $$

where ‖ ⋅ ‖ is the Euclidian norm, averaged on 30 independent runs. In each experiment, the following values are used to the initial conditions of analysed algorithms:

37 $$ \overset{\frown }{\mathbf{W}}(0)=0,\;P(0)=100\times \mathbf{I},\kern0.24em s(0)=1 $$

with **I** being the identity matrix of corresponding order.

### Time-varying parameter tracking in a stationary zero-mean white normal noise with possible outliers

**Fig. 2:**
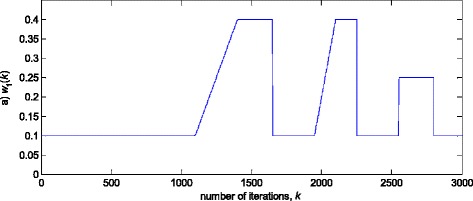
Time varying parameter trajectory *w*_1_(*k*) in (3)

**Fig. 3:**
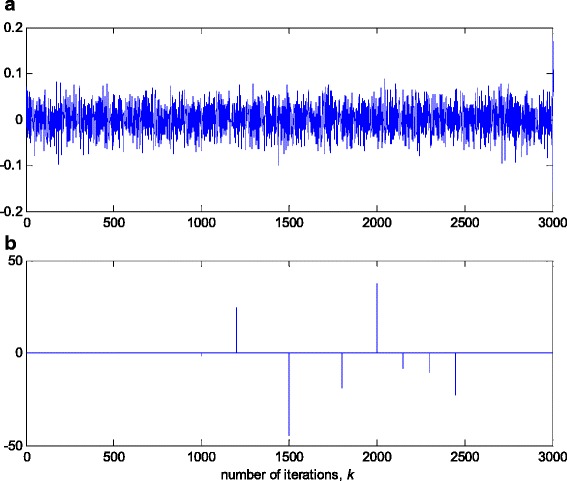
Realisation of additive zero-mean white noise *n*(*k*). **a** Pure zero-mean Gaussian samples (SNR = 25 dB). **b** Gaussian samples contaminated with outliers

**Fig. 4:**
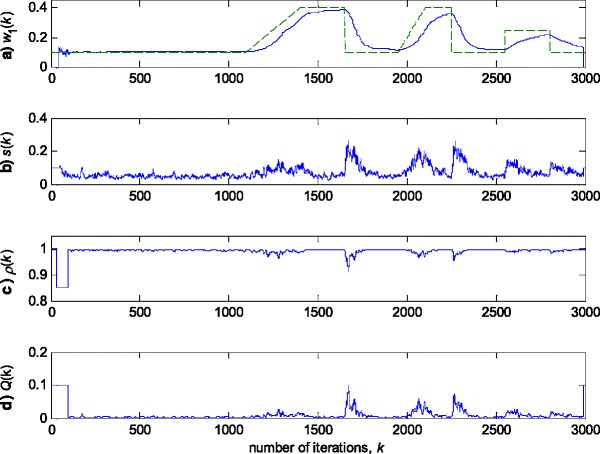
Experimental results for RRWLSV algorithm in zero-mean white Gaussian noise (see Fig. 3a). **a** Estimated (*solid line*) and true parameter (*dashed line*) trajectories (see Fig. 2). **b** Scale factor estimation, *s*(*k*). **c** VFF calculation, *ρ*(*k*). **d** Discrimination function calculation, *Q*(*k*)

**Fig. 5:**
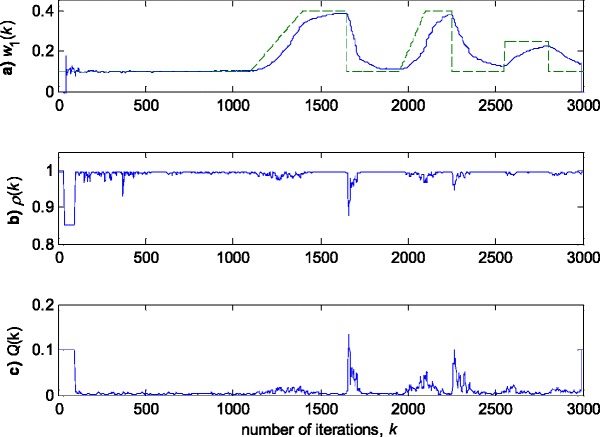
Experimental results for RLSVF algorithm in zero-mean Gaussian noise (Fig. 3a). **a** Estimated (*solid line*) and true parameter (*dashed line*) trajectories (see Fig. 2). **b** VFF calculation, *ρ*(*k*). **c** Discrimination function calculation, *Q*(*k*)

**Fig. 6:**
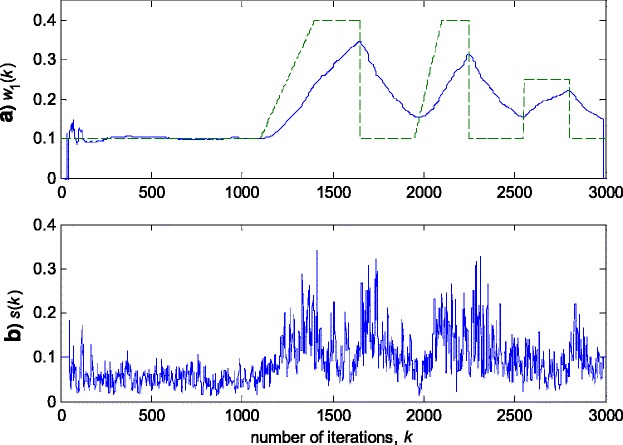
Experimental results for RRLSS algorithm in zero-mean Gaussian noise (Fig. 3a). **a** Estimated (*solid line*) and true parameter (*dashed line*) trajectories (Fig. 2). **b** Scale factor estimation, *s*(*k*)

**Fig. 7:**
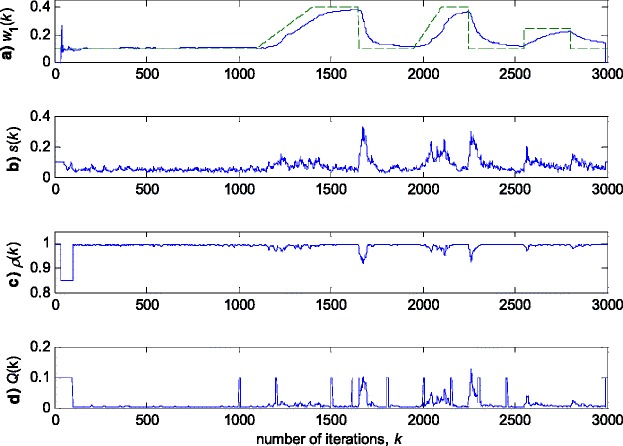
Experimental results for RRWLSV algorithm in zero-mean Gaussian noise contaminated by outliers (Fig. 3b). **a** Estimated (*solid line*) and true parameter (*dashed line*) trajectories (Fig. 2). **b** Scale factor estimation, *s*(*k*). **c** VFF calculation, *ρ*(*k*). **d** Discrimination function calculation, *Q*(*k*)

**Fig. 8:**
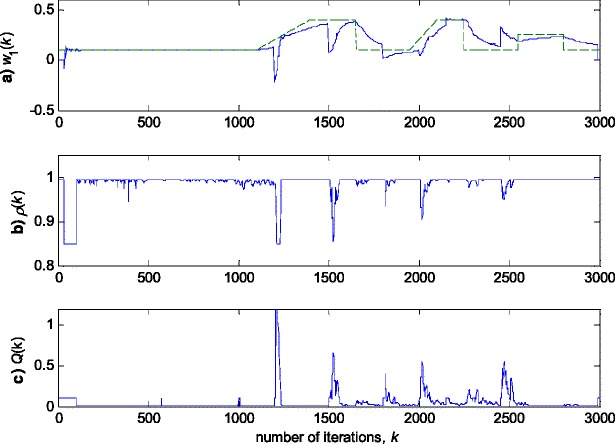
Experimental results for RLSVF algorithm in zero-mean Gaussian noise contaminated by outliers (Fig. 3b). **a** Estimated (*solid line*) and true parameter (*dashed line*) trajectories (Fig. 2). **b** VFF calculation, *ρ*(*k*). **c** Discrimination function calculation, *Q*(*k*)

**Fig. 9:**
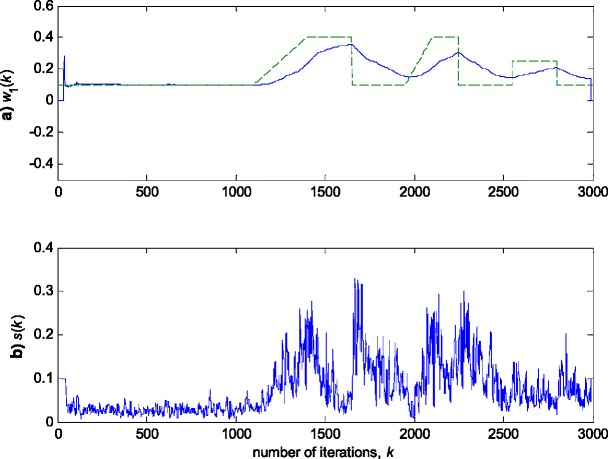
Experimental results for RRLSS algorithm in zero-mean Gaussian noise contaminated by outliers (depicted in Fig. 3b). **a** Estimated (*solid line*) and true parameter (*dashed line*) trajectories (Fig. 2). **b** Scale factor calculation, *s*(*k*)

**Fig. 10:**
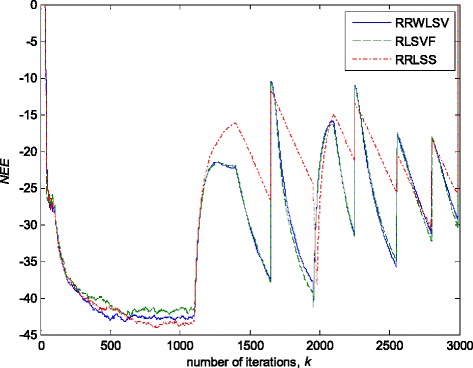
Normalised estimation errors *NEE* in (36), for different algorithms, in stationary zero-mean white normal Gaussian noise (SNR = 25dB); Experimental conditions are given in Figs. 2 and Fig 3a

**Fig. 11:**
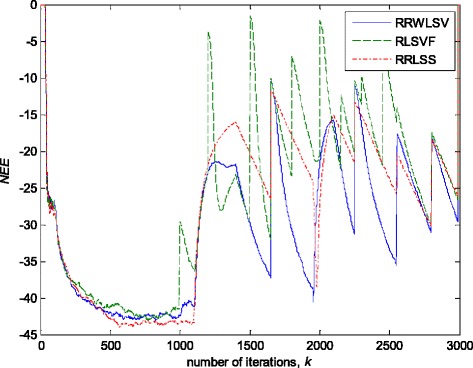
Normalised estimation errors *NEE* in (36 for different algorithms in stationary zero-mean white normal noise (SNR = 25 dB) contaminated by outliers; experimental conditions are given in Figs. 2 and Fig 3b

In this experiment, the first filter parameter *w*_1_ in (3) is changed using the trajectory depicted in Fig. 2. The variance of noise, *n*(*k*), is taken so that the SNR of 25 dB is achieved before the impulsive noise component is added. Figure 3 gives a realisation of the zero-mean white Gaussian sequence without (Fig. 3a) and with impulsive component (Fig. 3b), respectively. In Figs. 4, 5 and 6, the true and the estimated trajectories of changing parameter, under the pure zero-mean white Gaussian noise, are shown. Moreover, the estimated scale and variable forgetting factors are also depicted in these figures. Figures 7, 8 and 9 give the simulation results in the case of zero-mean and white and Gaussian noise with outliers. Figures 10 and 11 depicted the normalised estimation error in (36) obtained in the two discussed stationary noise environments, for the three analysed algorithms.

The obtained results have shown that the algorithms RRWLSV and RLSVF provide good and comparable results, due to the application of VFF in the weighted matrix update equation (see Eqs. 10, 12, 14 and 15), while the algorithm RRLSS gives bad parameter tracking performance, since it uses the fixed unit value of FF in (18), (see Figs. 4, 5, 6 and 10). Moreover, the results of simulations have indicated that the conventional RLS with VFF (RLSVF) is highly sensitive to outliers, since it does not use a robust influence function, *ψ*, in the parameter and weighted matrix update equations (see Eqs. 14 and 15). On the other hand, the RRLSS algorithm using scale factor estimation in (9), or combined the adaptive robustified scale and VFF factors (RRWLSV), are rather insensitive to outliers, due to the effect of robust influence function, *ψ*, in the parameter and weighted matrix update equations (Eqs. 17 and 18 or Eqs. 12 and 13). In addition, the algorithm RRWLSV has much better parameter tracking ability in the case of both time-varying parameters and impulsive noise environment than the algorithm RRLSS (see Figs. 7, 8, 9 and 11), due to the combined effects of scale factor and VFF (see Eqs. 12 and 13).

### Time-invariant parameter tracking in a non-stationary zero-mean normal noise with possible outliers

**Fig. 12:**
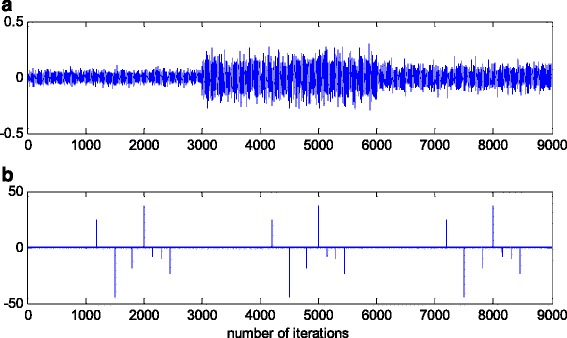
Realisation of additive noise, *n*(*k*). **a** Non-stationary zero-mean white normal noise samples with changing variances (SNR = 25, 15 and 20 dB). **b** Non-stationary zero-mean white normal noise samples, with changing variances, contaminated by outliers

In this experiment, the filter parameters are taken to be time invariant, but the noise variance is changed during the simulation, producing a non-stationary signal. The variance of additive noise, *n*(*k*), is taken so that for the three sequential signal segments, the SNRs of 25, 15 and 20 dB, respectively, are produced, before the impulsive noise component is added. In order to obtain better capture of long-term non-stationarity, in the sense of achieving good estimates of noise level changes, the scale factor, *s*, is averaged on the data frame of 400 samples. On the other hand, this will not affect the ability of VFF algorithm to track the filter parameter changes. Figure 12 shows a realisation of noise, without (Fig. 12a) and with (Fig. 12b) impulsive noise component.

**Fig. 13:**
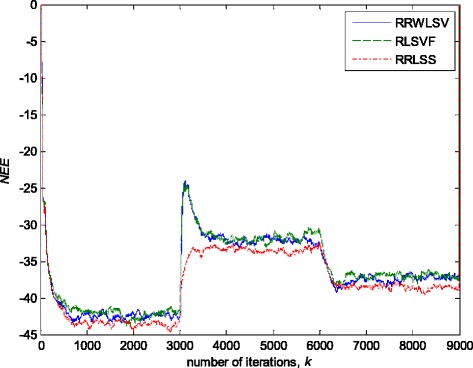
Normalised estimation error norm (*NEE*), for different algorithms in non-stationary Gaussian noise environment (Fig. 12a), and fixed parameters in (35)

**Fig. 14:**
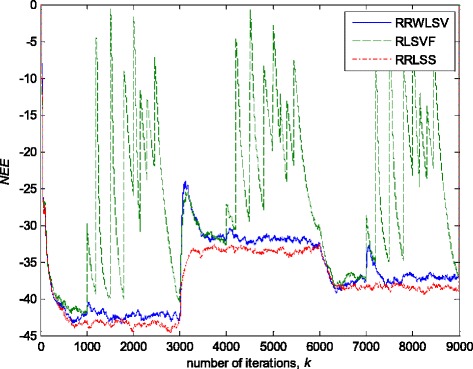
Normalised estimation error norm *NEE*, for different algorithms in non-stationary and impulsive noise environment (Fig. 12b), and fixed parameters in (35)

Figures 13 and 14 depict the obtained values of normalised estimation error (NEE) criterion in (36), without (Fig. 13) and with (Fig. 14) the presence of impulsive noise component.

In a pure zero-mean normal white noise environment (see, Fig. 12a), at the beginning of parameter estimation trajectory, all three discussed methods have similar behaviour (Fig. 13). Moreover, all three algorithms are rather insensitive to the changes of noise variance, due to the effects of scale factor estimations in (9) or (25), or VFF in (27)–(34) or (31)–(34), respectively, and give similar results at the whole parameter estimation trajectory, since there are no outliers (Fig. 13). The presented results in Fig. 14 indicated that the conventional RLS method with VFF (RLSVF) is highly sensitive to outliers, due to the lack of non-linear residual transformation, *ψ* (see Eqs. 14 and 15). On the other hand, the robust algorithms RRLSS and RRWLSV are rather insensitive to impulsive noise component, and give also comparable results, due to the effect of robust influence function, *ψ*, in the parameter and matrix update equations (see Eqs. 17 and 18 or Eqs. 12 and 13).

### Time-varying parameter tracking in a non-stationary zero-mean normal noise with possible outliers

**Fig. 15:**
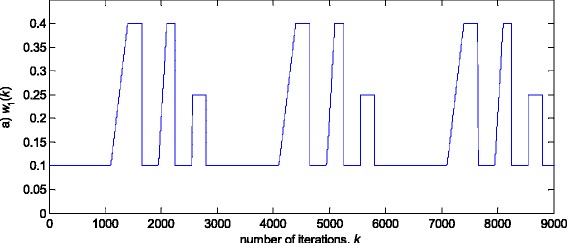
Time varying parameter trajectory, *w*_1_(*k*)

**Fig. 16:**
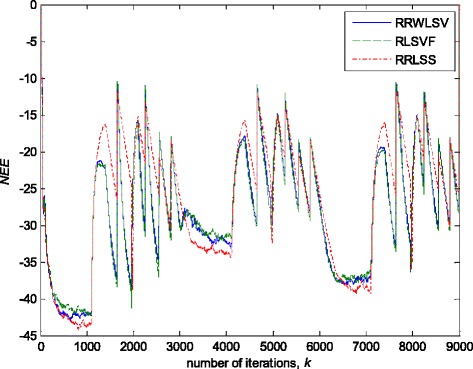
Normalised estimation error norm (*NEE*), for different algorithms, in non-stationary zero-mean Gaussian noise environment (Fig. 12a) and the time-varying parameter trajectory (Fig. 15)

**Fig. 17:**
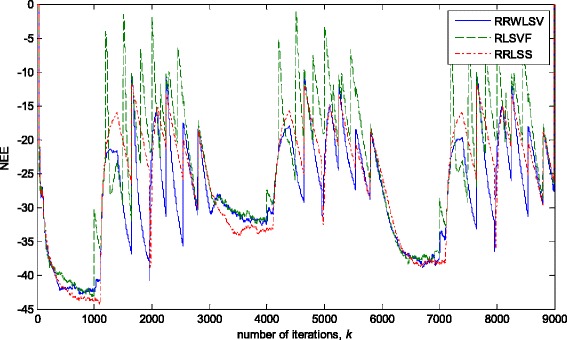
Normalised estimation error norm (*NEE*), for different algorithms, in non-stationary noise contaminated by outliers (Fig. 12b), and the time-varying parameter trajectory (Fig. 15)

In this experiment, the simulation scenario is the combination of the previous two examples, with the exception that the first filter parameter, *w*_1_, is adopted to be time-varying, using the parameters trajectory presented in Figs. 15, 16 and 17 depict the obtained *NEE* criterion (36), for different algorithms, without (Fig. 16) and with (Fig. 17) outliers, added to the non-stationary zero-mean normal noise with changing variance (see Fig. 12). The obtained results are in accordance to the conclusions derived from the previous two experiments.

In summary, the obtained results from the all three experiments have shown that, in order to properly estimate the time-varying parameters under the non-stationary and impulsive noise environment, both robustified VFF and adaptive M robust estimator with simultaneous estimation of parameters and scale factor (RRWLSV) are required. Moreover, an adequate non-linear residual transformation in the parameter update equation (Eq. 17 in RRLSS algorithm) is not sufficient for protecting well against the influence of outliers. Additionally, an important problem is also related to the way of recursive generation of the weighted matrix, **P**. It is found that the introduction of the Huber’s saturation type non-linearity, *ψ*, coupled with the proper decrease of the weighted matrix, **P**, depending on the non-linearly transformed residuals and VFF (Eqs. 12 and 13) provides low sensitivity to outliers and good parameter tracking performance (Figs. 4 and 7). The conventional M robust estimator (RRLSS) may converge very slowly, due to the introduction of the non-linearity first derivate, *ψ*′, and the unit FF in the recursive generation of the weighted matrix (Eq. 18). Namely, large residual realisations, *e*, make the decrease of the weighted matrix, **P**, very slow, since *ψ*′(*e*) = 0 in the saturation range (Eq. 18). This, in turn, results in an effect producing a slow convergence of parameter estimates (Figs. 6 and 9). The conventional RLS is also sensitive to outliers, but in a different way. In this algorithm, the weighted matrix, **P**, is not influenced by the residuals (Eq. 15). As a consequence, the fast decrease of **P**, independent of residuals, leads to the biased parameter estimates in the presence of outliers (see Fig. 8). In addition, the estimation of unknown noise variance (Eqs. 9 or 25) is essential to residual normalisation, in order to achieve a low sensitivity to the parameters defining the non-linear transformation of the prediction residuals in (4).

### Influence of outlier statistics

A real outlier statistics is not exactly known in practice, so a low sensitivity to outliers is very important for achieving the practical robustness. The effect of desensitising the parameter estimates related to the influence of outliers is illustrated in Tables 2 and 3, depicting the evaluation of sample mean and sample variance of the NEE statistics (36), for different outlier probability and intensity. It can be observed that the proposed algorithm automatically damp out the effects of outliers, and it is rather insensitive to various fractions of outliers (see Table 2), up to 15 %, as well as to high level outliers (see Table 3). For higher percentage of outliers, more than 20 %, the normal noise model contaminated by outliers is not adequate. Furthermore, *s*(*k*) is a nuisance parameter in the estimation of filter coefficients, as well as in the VFF computation. Its proper estimate is crucial for good performance of overall estimator, consisting of three coupled adaptive schemes. Since the complete algorithm performs quite well, this means that the estimation of scale factor also performs properly.

**Table 2:** The evaluation of the mean value and variance of *NEE*, for different outlier density, *ε* = *P*(*α*(*k*) = 1)

*ε*	0.01	0.05	0.10	0.15	0.20	0.25	0.3
NEE							
Mean value	−30.2529	−30.6593	−30.1427	−30.3464	−24.4299	−18.6086	−14.2801
Variance	92.9838	95.1626	111.2679	112.4948	226.8080	322.398	410.0782

**Table 3:** The evaluation of the mean value and variance of *NEE*, for different outlier intensity, ${\sigma}_e^2=\operatorname{var}A(k)$, where the nominal value is ${\sigma}_0^2={10}^4/12$

${\sigma}_e^2$	${10}^{-2}{\sigma}_0^2$	${10}^{-1}{\sigma}_0^2$	${\sigma}_0^2$	$10{\sigma}_0^2$	${10}^2{\sigma}_0^2$
NEE					
mean value	−30.8406	−30.3820	−30.4177	−30.4417	−30.3870
variance	94.2645	92.8128	94.9259	95.1020	94.6418

### Influence of initial conditions

The proposed RRWLSV algorithm, exposed in Table 1, is non-linear and, consequently, may be highly influenced by the initial conditions, **W**(0) and **P**(0) in (33), respectively. However, from the practical point of view, a low sensitivity to the initial conditions represents the desirable performance measure. Table 4 illustrates the influence of the initial condition **P**(0) to the estimation error.

**Table 4:** The evaluation of the mean square error norm, ‖**P**(*i*)‖, for different initial conditions (experimental conditions are the same as in the experiment 4.1)

**P**(0)	10^2^ **I**	10**I**	**I**	0.1**I**
Iterations				
50	366.9852	36.6985	0.3670	0.3530
100	0.9492	0.4582	1.1870	1.3091
1000	0.0100	0.0092	0.0085	0.0119

In general, large residual realisations in the initial steps, caused by large ‖**P**(0)‖, can make the decrease of ‖**P**(*i*)‖ very slow. This, in turn, may result in an undesirable effect producing a slow convergence of filter parameter estimates. However, the proposed RRWLSV algorithm is found to be relatively insensitive to the initial conditions, due to the proper way of generating of the weighting matrix, **P**(*i*), in (12) and (13), where the weighted term, *ω*(*i*) in (8), is used. This factor keeps the norm ‖**P**(*i*)‖ at values enough high for obtaining good convergence and, at the same time, enough small for preventing the described undesirable convergence effect. Similarly as in the experiment 4.1, it is observed that for higher values of ‖**P**(0)‖, the RRLSS algorithm converges very slowly. The reason lies in the introduction of the first derivative, *ψ*′, instead of the weighted term *ω* in (8), in the weighted matrix update equation, **P**(*i*), in (18), which is equal to zero in the saturation range. Thus, large residuals in the initial steps, caused by large ‖**P**(0)‖, make the decrease of ‖**P**(*i*)‖ very slow. This, in turn, results in the described cumulative effect, producing a slow convergence of parameter estimates (see Table 4).

### Influence of model order

**Fig. 18:**
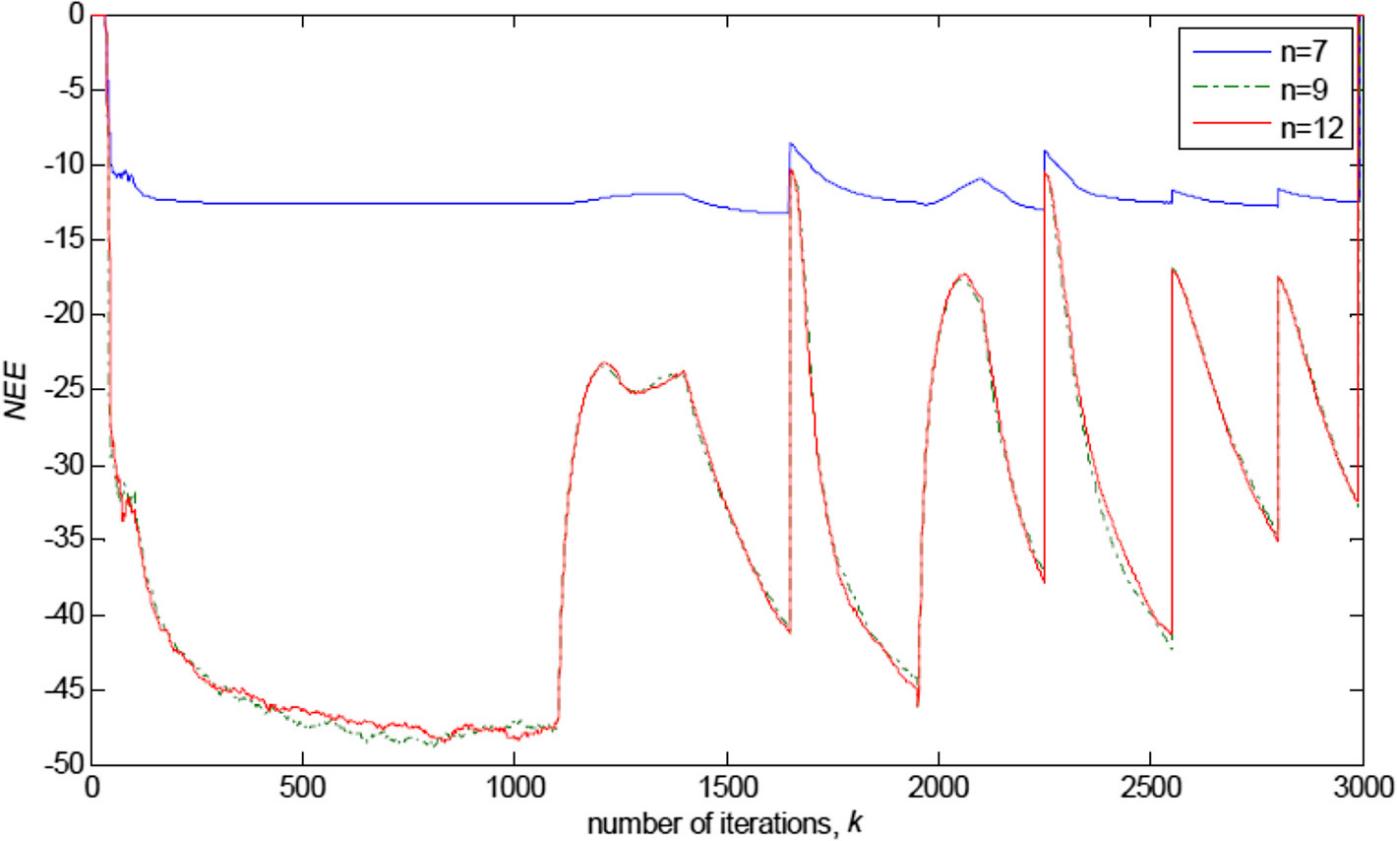
Normalised estimation error norm (*NEE*) in (36), for the experimental conditions described in 4.1 (Figs. 2 and 3b) and the algorithm RRWLSV, in the cases of lower (*n* = 7), higher (*n* = 12) and exact (*n* = 9) model order

The selection of model structure depends on the intended model application [31]. With the adopted FIR model structure, one has to select the order, *n*, of the parameter vector, **W** in (3). The model order should not be selected too low, since then, all system dynamics cannot be described properly. However, it should not be selected too high either, since the higher the model order, the most parameters need to be estimated and the higher the variance of the parameter estimates is. Thus, increasing the model order beyond the true order of the system will not add to the quality of the model. Hence, the model order is some sort of compromise between fitting the data and model complexity [32, 33]. Figure 18 illustrates the discussion through the obtained *NEE* criterion in (36), for the experimental conditions described in 4.1, in the cases of lower (*n* = 7), higher (*n* = 12) and exact (*n* = 9) model order of the FIR system.

### Influence of additive noise correlations

**Fig. 19:**
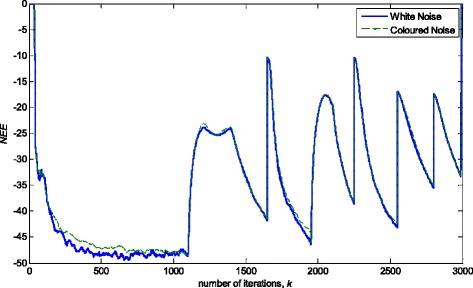
Normalised estimation error norm (*NEE*) in (36), for the experimental conditions in the case of white and coloured noise (see Fig. 20)

**Fig. 20:**
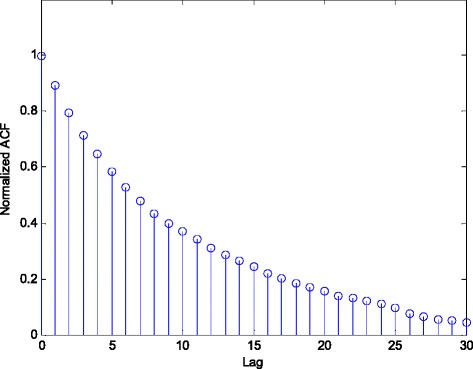
Normalised autocorrelation function of coloured noise

The FIR model structure does not take into account the coloured additive noise. However, in general, additive noise, *n*(*k*), may represent any kind of noise, with arbitrary colour. Such noise can be simulated by filtering a zero-mean white noise through a linear time-invariant (LTI) system [34, 35]. In this way, one can shape the fit of a time-invariant model under a stationary noise environment to the frequency domain. It can be shown that, in general case, the resulting estimate is a compromise between fitting the estimated constant model parameters to the true one and fitting the noise model spectrum to the prediction error spectrum [35]. In addition, the estimates can be improved by using prefilters. The analysis is based on the limit parameter value to which the parameter estimates converge asymptotically and corresponding limit criterion [35]. However, such an analysis is not possible on the short data sequences, whether the situation is stationary or not. Therefore, the answer to the question concerning the insensitivity to noise statistics, as well as the other questions related to the practical robustness, can be obtained only by simulations. Figure 19 depicts the obtained *NEE* criterion in (36), with the experimental conditions exposed in 4.1, but using the coloured noise with the given autocorrelation function in Fig. 20, [36]. The experimental results indicate that the proposed RRWLSV filtering algorithm copes satisfactorily with the coloured noise.

It should be noted that in a FIR model structure, the noise model is fixed and equal to the unit constant.

## Conclusions

The estimation problem of time-varying adaptive FIR filter parameters in the situations characterised by non-stationary and impulsive noise environments has been discussed in the article. The posed problem is solved efficiently by application of a new adaptive robust algorithm, including a combination of the M robust concept, extended to the estimation of both filter parameters and unknown noise variance simultaneously, and adaptive robustified variable forgetting factor. The variable forgetting factor is determined by linear mapping of a suitably defined robust discrimination function, representing the ratio of robustified extended prediction error criterion, using M robust approach, and M robust type recursive estimate of noise variance. Since the robustified version of extended prediction error criterion is calculated on sliding data frame of proper length, it represents a robust measure of local data non-stationarity. On the other hand, the total noise variance robust recursive estimate is rather insensitive to the local non-stationarity effects, so that the adopted robust discrimination function represents a suitable normalised robust measure of the degree of signal non-stationarity. In addition, since the total noise variance, or the so-called scale factor, and variable forgetting factor are adaptively calculated with respect to the prediction errors, the proposed algorithm works properly in stationary and non-stationary situations with possible outliers. Simulation results have shown that the new method gives higher accuracies of parameter estimates, and ensures better parameter tracking ability, in comparison to the conventional least-squares algorithm with variable forgetting factor, and the standard M robust algorithm with scale factor estimation. Moreover, the standard least-squares algorithm with variable forgetting factor is very sensitive to outliers, while the new adaptive robust method with combined scale and variable forgetting factor, and the conventional M robust-based method, with scale factor, are rather insensitive to such a disturbance. However, the proposed adaptive M robust algorithm with combined scale and forgetting factors has much better parameter tracking performance than the conventional M robust algorithm with only scale factor. The experimental analysis has shown that the real practical robustness and good tracking performances are connected with both the non-linear transformation of predictionresiduals and an adequate recursive generation of the weighted matrix, depending on the non-linearity form and variable forgetting factor. Moreover, a recursive estimation of the unknown noise variance is essential for defining properly the non-linearity form. In summary, in order to properly estimate the time-varying parameters under the non-stationary and impulsive noise, both robustified variable forgetting factor and simultaneous adaptive M robust estimation of system parameters and unknown noise variance are required.

The proposed adaptive M robust estimators for generating scale factor and variable forgetting factor are general, while the adaptive M robust parameter estimation procedure depends on assumed signal, or system, model structure. Moreover, it can be easily applied to the other commonly used signal, or system models, including AR, ARX, ARMA and ARMAX models, respectively, or even non-linear models. Furthermore, the proposed adaptive robustified algorithm is the one that, with proper adaptation, can be used as a robust alternative to the conventional recursive least squares or Kalman filter, respectively. These applications arise in various fields, including speech processing, biomedical signal processing, image analysis, and failure detection in measurement and control.

## Appendix 1

### Derivation of relations (10)–(13).

The solution of (6) is given by

38 $$ \widehat{\mathbf{W}}(k)={\mathbf{R}}^{-1}(k){\displaystyle \sum_{i=1}^k\beta \left(k,i\right)\mathbf{X}(i)d(i)} $$

where

39 $$ \beta \left(k,i\right)={\rho}^{k-i}\omega (i) $$

with *ω*(*i*) being defined by (8) when **W**_0_ is replaced by **Ŵ**(*k* − 1). Moreover, *e*(*i*, **W**) in (2) is defined by (11) with **W** = **Ŵ**(*k* − 1), while the matrix **R** in (38) is given by

40 $$ \mathbf{R}(k)={\displaystyle \sum_{i=1}^k\beta}\left(k,i\right)\mathbf{X}(i){\mathbf{X}}^T(i)=\rho {\displaystyle \sum_{i=1}^{k-1}\beta}\left(k-1,i\right)\mathbf{X}(i){\mathbf{X}}^T(i)+\omega (k)\mathbf{X}(k){\mathbf{X}}^T(k) $$

from which it follows

41 $$ \mathbf{R}(k)=\rho \mathbf{R}\left(k-1\right)+\omega (k)\mathbf{X}(k){\mathbf{X}}^T(k) $$

Furthermore, one can also write

42 $$ {\displaystyle \sum_{i=1}^k\beta \left(k,i\right)\mathbf{X}(i)d(i)}=\rho {\displaystyle \sum_{i=1}^{k-1}\beta \left(k-1,i\right)\mathbf{X}(i)d(i)}+\omega (k)\mathbf{X}(k)d(k) $$

Taking into account (38), one concludes from (42)

43 $$ {\displaystyle \sum_{i=1}^k\beta \left(k,i\right)\mathbf{X}(i)d(i)}=\rho \mathbf{R}\left(k-1\right)\widehat{\mathbf{W}}\left(k-1\right)+\omega (k)\mathbf{X}(k)d(k) $$

Furthermore, by calculating *ρ***R**(*k* − 1) from (41), and replacing it into (43), one obtains

44 $$ {\displaystyle \sum_{i=1}^k\beta \left(k,i\right)\mathbf{X}(i)d(i)}=\left[\mathbf{R}(k)-\omega (k)\mathbf{X}(k){\mathbf{X}}^T(k)\right]\widehat{\mathbf{W}}\left(k-1\right)+\omega (k)\mathbf{X}(k)d(k) $$

Finally, by replacing (44) into (38), one obtains the relations (10) and (11) with the gain matrix

45 $$ \mathbf{K}(k)={\mathbf{R}}^{-1}(k)\omega (k)\mathbf{X}(k) $$

In addition, by introducing the weighting matrix **P**(*k*) = **R**^− 1^(*k*), it follows from (41)

46 $$ \mathbf{P}(k)={\left[\rho\;{\mathbf{P}}^{-1}\left(k-1\right)+\omega (k)\mathbf{X}(k){\mathbf{X}}^T(k)\right]}^{-1} $$

After applying the well-known matrix inversion lemma [25, 26]

47 $$ {\left(\mathbf{A}+\mathbf{B}\mathbf{C}\mathbf{D}\right)}^{-1}={\mathbf{A}}^{-1}-{\mathbf{A}}^{-1}\mathbf{B}{\left({\mathbf{C}}^{-1}+\mathbf{D}{\mathbf{A}}^{-1}\mathbf{B}\right)}^{-1}\mathbf{D}{\mathbf{A}}^{-1} $$

on (46) with **A** = *ρ***P**^− 1^(*k* − 1), **B** = **X**(*k*), **C** = *ω*(*k*) and **D** = **X**^*T*^(*k*), one concludes from (47)

48 $$ \mathbf{P}(k)=\frac{1}{\rho}\left\{\mathbf{P}\left(k-1\right)-\frac{\mathbf{P}\left(k-1\right)\mathbf{X}(k){\mathbf{X}}^T(k)\mathbf{P}\left(k-1\right)}{\rho {\omega}^{-1}(k){\mathbf{X}}^T(k)\mathbf{P}\left(k-1\right)\mathbf{X}(k)}\right\} $$

By substituting **R**^− 1^(*k*) = **P**(*k*) from (48) into (45), one can write further

49 $$ \mathbf{K}(k)=\frac{\mathbf{P}\left(k-1\right)\mathbf{X}(k)\omega (k)}{\rho +{\mathbf{X}}^T(k)\mathbf{P}\left(k-1\right)\mathbf{X}(k)\omega (k)} $$

Finally, by introducing **M**(*k*) from (12), and substituting (49) into (48), one obtains the relations (12) and (13), which completes the proof.

## Appendix 2

### Derivation of relations (24)–(25).

Starting from (21)–(23), one can write

50 $$ \begin{array}{c}k{J}_k\left(s/\widehat{\mathbf{W}}\right)= \ln (s)+\left(k-1\right){J}_{k-1}\left(s/\widehat{\mathbf{W}}\right)+f\left(\frac{e\left(k,\widehat{\mathbf{W}}\right)}{s}\right)\\ {}\approx \ln (s)+f\left(\frac{e\left(k,\widehat{\mathbf{W}}\right)}{s}\right)\end{array} $$

and

51 $$ k\frac{\partial {J}_k\left(s/\widehat{\mathbf{W}}\right)}{\partial s}=\frac{1}{s}-\frac{e\left(k,\widehat{\mathbf{W}}\right)}{s^2}g\left(\frac{e\left(k,\widehat{\mathbf{W}}\right)}{s}\right) $$

where *g*(⋅) = *f* ' (⋅). Furthermore, one can also write from (19)

52 $$ \frac{\partial J\left(\sigma /\widehat{\mathbf{W}}\right)}{\partial \sigma }=\frac{1}{\sigma }-E\left\{\frac{e\left(k,\widehat{\mathbf{W}}\right)}{\sigma^2}g\left(\frac{e\left(k,\widehat{\mathbf{W}}\right)}{\sigma}\right)\right\} $$

and

53 $$ \frac{\partial^2J\left(\sigma /\widehat{\mathbf{W}}\right)}{\partial {\sigma}^2}=-\frac{1}{\sigma^2}+E\left\{g\hbox{'}\left(\frac{e\left(k,\widehat{\mathbf{W}}\right)}{\sigma}\right)\frac{e^2\left(k,\widehat{\mathbf{W}}\right)}{\sigma^4}\right\}+\frac{2}{\sigma }E\left\{\frac{e\left(k,\widehat{\mathbf{W}}\right)}{\sigma^2}g\left(\frac{e\left(k,\widehat{\mathbf{W}}\right)}{\sigma}\right)\right\} $$

In the vicinity of the optimal solution, one concludes ∂*J*(*σ*/**Ŵ**)/∂*σ* = 0 in (52), from which it follows

54 $$ E\left\{\frac{e\left(k,\widehat{\mathbf{W}}\right)}{\sigma^2}g\left(\frac{e\left(k,\widehat{\mathbf{W}}\right)}{\sigma}\right)\right\}=\frac{1}{\sigma } $$

Furthermore, by substituting (54) in (53), one obtains

55 $$ \frac{\partial^2{J}_k\left(s/\widehat{\mathbf{W}}\right)}{\partial {s}^2}\approx \frac{\partial^2J\left(s/\widehat{\mathbf{W}}\right)}{\partial {\sigma}^2}=\frac{a}{s^2} $$

where the constant

56 $$ a=1+E\left\{g\hbox{'}\left(\frac{e\left(k,\widehat{\mathbf{W}}\right)}{s}\right)\frac{e^2\left(k,\widehat{\mathbf{W}}\right)}{s^2}\right\}\approx 1 $$

Finally, if one substitutes (51), (55) and (56) into (20), one obtains an approximate optimal solution for the scale factor estimation in the recursive form (24). In addition, if we assume for the moment that *s*(*k* − 1) is close to *s*(*k*), and take into account the relation (8), the expression (24) can be rewritten in the alternative form (25), which completes the proof.

## Appendix 3

### The derivation of mean value and variance of the random variable generating the outliers

The outliers *n*(*k*) for each time step *k* are generated by the random variable (r.v.) *n* = *αA*, representing the function of two variables, where *α* is the discrete binary r.v. taking the values *α*_1_ = 0 with the probability *p*_1_ = *P*{*α* = *α*_1_}, and *α*_2_ = 1 with the probability *p*_2_ = *P*{*α* = *α*_2_}, *p*_1_ + *p*_2_ = 1. Moreover, *A* is the continuous zero-mean r.v. with variance ${\sigma}_A^2$, and it is supposed that *α* and *A* are independent. The mean *E*{*n*} can be determined directly in terms of the joint p.d.f. *f*_*α*,*A*_(*α*, *A*) without the need for evaluating p.d.f. of *n*, that is [35]

57 $$ E\left\{n\right\}=E\left\{\alpha A\right\}={\displaystyle \underset{-\infty }{\overset{\infty }{\int }}{\displaystyle \underset{-\infty }{\overset{\infty }{\int }}\alpha A{f}_{\alpha A}\left(\alpha, A\right) d\alpha dA}}=E\left\{\alpha \right\}E\left\{A\right\}={m}_{\alpha }{m}_A $$

since *α* and *A* are independent, yielding *f*_*αA*_(*α*, *A*) = *f*_*α*_(*α*)*f*_*A*_(*A*). However, due to the fact that *m*_*A*_ = *E*{*A*} = 0, one concludes *m*_*n*_ = *E*{*n*} = 0. The variance of r.v. *n* is given by

58 $$ {\sigma}_n^2=E\left\{{n}^2\right\}-{E}^2\left\{n\right\}={m}_{2,n}-{m}_n^2 $$

where *m*_2,*n*_ and *m*_*n*_ are non-centralized moments of the second and the first order, respectively. Similarly as before, the variance can be determined as

59 $$ {\sigma}_n^2={\sigma}_{\alpha A}^2=E\left\{{\left(\alpha A\right)}^2\right\}-{E}^2\left\{\alpha A\right\} $$

that is, since *E*{*αA*} = 0

60 $$ {\sigma}_{\alpha A}^2=E\left\{{\alpha}^2{A}^2\right\}={\displaystyle \underset{-\infty }{\overset{\infty }{\int }}{\displaystyle \underset{-\infty }{\overset{\infty }{\int }}{\alpha}^2{A}^2{f}_{\alpha A}\left(\alpha, A\right) d\alpha dA}}={m}_{2,\alpha }{m}_{2,A} $$

Here, ${m}_{2,\alpha }={\sigma}_A^2$, since *m*_*A*_ = 0, while

61 $$ {m}_{2,\alpha }={\alpha}_1^2{p}_1+{\alpha}_2^2{p}_2=0{p}_1+1{p}_2={p}_2 $$

from which it follows

62 $$ {\sigma}_n^2={p}_2{\sigma}_A^2;\kern0.24em {p}_2=P\left\{\alpha =1\right\}=\varepsilon $$

where *ε* is the density or fraction of outliers.

## Abbreviations

EPE: extended prediction error
FIR: finite impulse response
FF: forgetting factor
FFF: fixed value of forgetting factor
LS: least squares
M: approximate maximum-likelihood
MAD: median of absolute median deviations
ML: maximum-likelihood
*n*: parameter vecor order
*N*: memory length
NEE: normalised estimation error
PA: parallel adaptation
*Q*: discrimination function
RLS: recursive least squares
RLSVF: recursive least squares with variable forgetting factor
RRLS: robustified recursive least squares
RRLSS: robustified recursive least squares with scale factor
RRWLSV: robustified recursive weighted least squares with both scale and variable forgetting factors
SNR: signal-to-noise ratio
VFF: variable forgetting factor
WLS: weighted least-squares
